# A New Zamilon-like Virophage Partial Genome Assembled from a Bioreactor Metagenome

**DOI:** 10.3389/fmicb.2015.01308

**Published:** 2015-11-27

**Authors:** Meriem Bekliz, Jonathan Verneau, Samia Benamar, Didier Raoult, Bernard La Scola, Philippe Colson

**Affiliations:** ^1^URMITE, UM 63, Centre National de la Recherche Scientifique 7278, IRD 198, INSERM U1095, Aix-Marseille UniversityMarseille, France; ^2^IHU Méditerranée Infection, Pôle des Maladies Infectieuses et Tropicales Clinique et Biologique, Fédération de Bactériologie-Hygiène-Virologie, Centre Hospitalo-Universitaire TimoneMarseille, France; ^3^Special Infectious Agents Unit, King Fahd Medical Research Center, King Abdulaziz UniversityJeddah, Saudi Arabia

**Keywords:** virophage, Zamilon, metagenome, giant virus, mimivirus, *Megavirales*, nucleocytoplasmic large DNA virus, bioreactor

## Abstract

Virophages replicate within viral factories inside the *Acanthamoeba* cytoplasm, and decrease the infectivity and replication of their associated giant viruses. Culture isolation and metagenome analyses have suggested that they are common in our environment. By screening metagenomic databases in search of amoebal viruses, we detected virophage-related sequences among sequences generated from the same non-aerated bioreactor metagenome as recently screened by another team for virophage capsid-encoding genes. We describe here the assembled partial genome of a virophage closely related to Zamilon, which infects *Acanthamoeba* with mimiviruses of lineages B and C but not A. Searches for sequences related to amoebal giant viruses, other *Megavirales* representatives and virophages were conducted using BLAST against this bioreactor metagenome (PRJNA73603). Comparative genomic and phylogenetic analyses were performed using sequences from previously identified virophages. A total of 72 metagenome contigs generated from the bioreactor were identified as best matching with sequences from *Megavirales* representatives, mostly *Pithovirus sibericum*, pandoraviruses and amoebal mimiviruses from three lineages A–C, as well as from virophages. In addition, a partial genome from a Zamilon-like virophage, we named Zamilon 2, was assembled. This genome has a size of 6716 base pairs, corresponding to 39% of the Zamilon genome, and comprises partial or full-length homologs for 15 Zamilon predicted open reading frames (ORFs). Mean nucleotide and amino acid identities for these 15 Zamilon 2 ORFs with their Zamilon counterparts were 89% (range, 81–96%) and 91% (range, 78–99%), respectively. Notably, these ORFs included two encoding a capsid protein and a packaging ATPase. Comparative genomics and phylogenetic analyses indicated that the partial genome was that of a new Zamilon-like virophage. Further studies are needed to gain better knowledge of the tropism and prevalence of virophages in our biosphere and in humans.

## Introduction

Mimivirus was the first amoeba-associated giant virus described in 2003 (La Scola et al., 2003). This virus founded a new viral family, *Mimiviridae*, and led, through culturing on *Acanthamoeba* spp., to the subsequent isolation of dozens of relatives, and members from new families or putative families of giant viruses (Pagnier et al., 2013; Reteno et al., 2015). Three lineages, named A, B, and C, were described for amoebal mimiviruses (Colson et al., 2012), and distant mimiviruses with marine phagocytic protistan hosts were also described (Fischer et al., 2010; Yutin et al., 2013a).

Mimivirus discovery further led to the discovery of ≈50–60 nm-large icosahedral viruses that co-infect *Acanthamoeba* spp. with mimiviruses (La Scola et al., 2008). These viruses replicate within mimivirus viral factories, and decrease the infectivity and replication of their associated giant virus while inducing morphological defects in mimivirus virions; these were named virophages due to their functional analogy with bacteriophages (La Scola et al., 2008; Desnues et al., 2012a). Thus far, five virophages have been isolated from *Acanthamoeba* spp. in water samples collected from distinct geographical locations (La Scola et al., 2008; Fischer and Suttle, 2011; Gaia et al., 2013, 2014) (Table 1). Their genomes are 17.2–26.4 kilobase pairs (kbp) in length and encode 16–24 proteins. Sputnik was the first virophage isolated in 2008, from cooling tower water collected in Paris (La Scola et al., 2008). Then, Sputnik 2 and 3, whose genomes are very similar to Sputnik, were isolated with other amoebal mimiviruses of lineage A (Desnues et al., 2012b; Gaia et al., 2013). More recently, Zamilon was co-isolated from Tunisian soil with Mont1 virus, an amoebal mimivirus of lineage C (Gaia et al., 2014). Noteworthy, this new virophage differed by its ability to replicate with mimiviruses of lineages B-C but not A. Other virophages were described with distant mimiviruses. Thus, Mavirus was isolated with *Cafeteria roenbergensis* virus in a bi-flagellate protozoan host from the coastal waters of Texas, USA (Fischer and Suttle, 2011), and the genome of a virophage associated with *Phaeocystis globosa* virus (PgV)-16T was retrieved from Dutch coastal waters (Santini et al., 2013). In addition, 12 complete or partial genomic sequences of virophages were assembled from environmental metagenomes (Table 1). The Organic Lake virophage (OLV) genome was obtained from a hypersaline lake in Antarctica, concurrently with those from distant mimiviruses initially classified as phycodnaviruses (Yau et al., 2011). Eight additional genome sequences from virophages named YSLV1-7 and ALM were retrieved from Yellowstone Lake, USA, and Ace Lake, Antarctica, respectively (Zhou et al., 2013, 2015). Five of these virophages (YSLV1-4 and 6) were related to OLV, ALM was related to Mavirus and YSLV5 and 7 represented a novel lineage. Virophage-related sequences were also detected in metagenomes generated from Ace Lake in the Antarctic, a freshwater lake in Panama, a hypersaline lagoon and an ocean upwelling in the Galapagos, and Delaware Bay estuary, USA (Yau et al., 2011), and Sputnik virophage-related sequences were identified in the metaviromes of two Antarctic hyperarid desert soil communities (Zablocki et al., 2014). Finally, Yutin et al. recently identified major capsid proteins (MCPs) from putative virophages in metagenomes generated from sheep rumen and freshwater sediment, marine water, wastewater, activated sludge, and a bioreactor (Yutin et al., 2015). Phylogeny reconstruction showed that MCPs from activated sludge and bioreactor metagenomes were clustered with Sputnik/Zamilon virophages, whereas those from Sargasso sea metagenome were clustered with Organic Lake/Yellowstone Lake virophages.

**Table 1:** **Main features of currently discovered virophages**.

**Virophage**	**GenBank accession no**.	**Year of description**	**Geographical location**	**Associated giant virus**	**Protistan host**	**Discovery tool**	**Genome**			**References**
							**Size (pb)**	**Number of ORFs**	**G+C (%)**	
Sputnik	EU606015	2008	Cooling tower water, Paris, France	Mamavirus	*Acanthamoeba polyphaga*	Culture	18,343	21	27	La Scola et al., 2008
Mavirus	NC_015230	2010	Coastal waters, Texas, USA	*Cafeteria roenbergensis*	*Marine phagotrophic*	Culture	19,063	20	30	Fischer and Suttle, 2011
				virus	flagellate					
OLV	HQ704801	2011	Organic Lake, Antarctica	Organic lake, phycodnavirus (distant mimivirus)	Prasinophytes	Genomic	26,421	24	37	Yau et al., 2011
Sputnik 2	NC_023846	2012	Lens liquid, Marseille, France	Lentillevirus	*Acanthamoeba polyphaga*	Culture	18,338	20	27	Desnues et al., 2012b
Sputnik 3	NC_023847	2013	Soil, France	Mamavirus	*Acanthamoeba polyphaga*	Culture	18,338	20	27	Gaia et al., 2013
*Phaeocystis globosa* virophage	NC_021333	2013	Dutch coastal waters, southern North Sea	*Phaeocystis globosa virus (PgV)-16T* (distant mimivirus)	Algae	Genomic	19,527	16	36	Santini et al., 2013
YSLV1	KC556924	2013	Yellowstone Lake, USA	N.d.	N.d.	Metagenomics	27,849	26	33	Zhou et al., 2013
YSLV2	KC556925	2013	Yellowstone Lake, USA	N.d.	N.d.	Metagenomics	23,184	21	34	Zhou et al., 2013
YSLV3	KC556926	2013	Yellowstone Lake, USA	N.d.	N.d.	Metagenomics	27,05	23	35	Zhou et al., 2013
YSLV4	KC556922	2013	Yellowstone Lake, USA	N.d.	N.d.	Metagenomics	28,306	34	37	Zhou et al., 2013
ALM	KC556923	2013	Ace Lake, Antarctica	N.d.	N.d.	Metagenomics	17,767	22	27	Zhou et al., 2013
Zamilon	NC_022990	2014	Tunisia	Mont1 (mimivirus)	*Acanthamoeba polyphaga*	Culture	17,276	20	30	Gaia et al., 2014
YSLV5	KM502589	2014	Yellowstone Lake, USA	N.d.	N.d.	Metagenomics	29,767	32	51	Zhou et al., 2013
YSLV6	KM502590	2014	Yellowstone Lake, USA	N.d.	N.d.	Metagenomics	24,837	29	27	Zhou et al., 2013
YSLV7	KM502591	2014	Yellowstone Lake, USA	N.d.	N.d.	Metagenomics	23,193	26	27	Zhou et al., 2013

*ALM, Ace Lake Mavirus; bp, pase pairs; N.d., not determined; OLV, Organic Lake virophage; ORF, open reading frame; YSLV, Yellowstone Lake virophage*.

Virophages have been revealed as important biological entities in evolutionary biology. Thus, the Sputnik genome was described as a mosaic encoding genes linked to viruses infecting representatives from *Eukarya, Archaea*, and *Bacteria* (La Scola et al., 2008). Additionally, virophages are a component of the mimivirus mobilome, being capable of integrating as provirophages, and may be involved in nucleic sequence exchanges between giant viruses and other microbes within amoebae (La Scola et al., 2008; Desnues et al., 2012b), and the Mavirus virophage showed an evolutionary relationship with Polinton (or Maverick) transposons present in various eukaryotes (Fischer and Suttle, 2011; Yutin et al., 2013b). Besides, Yutin et al. recently described virophage-Polinton chimeric genomes (Yutin et al., 2015).

By screening various available environmental metagenomic databases in search of giant viruses and virophages, we detected several robust matches with sequences from the Zamilon virophage, and, concurrently, mimiviruses and other amoebal giant viruses, among sequences from the same bioreactor metagenome as recently explored by Yutin et al. (2015). Whereas, this team detected strong homologies to the Zamilon capsid gene in this metagenome, our first-line approach consisted in a broader search for any sequence matching with virophages, and not only sequences matching capsid proteins. This allowed us to map sequences to 15 genes from the Zamilon genome. We report here the assembly and analyses of the partial genome sequence of a novel Zamilon-like virophage (we named Zamilon 2) from this metagenome.

## Materials and methods

### Metagenome analyzed

A non-aerated poplar wood chips Bioreactor metagenome was downloaded from the NCBI GenBank genome sequence database (GenBank Accession number: AGTN00000000.1; BioSample: SAMN02954264; Bioproject: PRJNA73603) (Van der Lelie et al., 2012). This terrestrial environmental biome metagenome had been generated from poplar woody material in New York State, USA (Van der Lelie et al., 2012). Metagenomic sequences had been obtained from non-sterile yellow poplar sawdust taken from the inside of a pile, then placed in a plastic bucket, humidified with MgSO_4_ solution and incubated at 30°C in a micro-aerobic/anaerobic atmosphere for 12 months; sequencing had been performed on DNA isolated from biomass and liquid from the anaerobic zone. The bioreactor DNA had been submitted to 454 Roche and Sanger sequencing, and 748,672 contig sequences had been generated by whole genome shotgun sequencing (WGS) WGS from this bioreactor sample. All these analyses were described in the study by Van der Lelie et al. (2012).

### Dataset of amino acid sequences from *Megavirales* members, virophages, and *Acanthamoeba castellanii* used as queries

Query sequences used to search for related sequences among the WGS dataset from the metagenome were amino acid sequences from the members of the proposed order *Megavirales*, including poxviruses, phycodnaviruses, asfarviruses, ascoviruses, iridoviruses, mimiviruses, marseilleviruses, *Pandoravirus salinus* and *P. dulcis, Pithovirus sibericum*, and from virophages (Table 1). Most of these sequences were available from the NCBI GenBank sequence databases (http://www.ncbi.nlm.nih.gov/genbank/). In addition, other mimivirus and marseillevirus sequences, not publicly available and corresponding to unpublished genomes whose sequence was obtained through high-throughput sequencing at our research unit, were used for the analyses. Sputnik 2 and 3 virophage gene contents were excluded from the analysis because they are nearly identical to that of the original Sputnik strain. We also searched for metagenome sequences related to the amoebal host for giant viruses and virophages by using amino acid sequences from *Acanthamoeba castellanii* downloaded from GenBank.

### Search strategy for virus and amoeba-related metagenome sequences

Sequences related to *Megavirales* representatives, virophages and *A. castellanii* were searched for using BLASTx (with an *e* ≤ 10^−3^) in the non-aerated bioreactor metagenome WGS dataset (Altschul et al., 1997). The sequences showing significant BLASTx hits to amoebal virus genes and those of their host were sorted and used for further analyses. Thus, these sequences were then compared to the NCBI GenBank non-redundant protein sequence database (nr) and sequences related to *Megavirales* representatives only available from the URMITE sequence database using BLASTx and BLASTn with default parameters, in order to eliminate false positive matches with other organisms.

### *De novo* metagenomic assembly and prediction of virophage-encoded genes

Contig sequences corresponding to virophages were assembled after mapping onto the genomes of virophages for which best BLASTx hits had been obtained, and visualized using the CLC Genomics version 6.0.1 software (http://www.clcbio.com/) with standard options. A consensus genome sequence was generated using the CLC Genomics software. Guanine and Cytosine (G+C) content of this genome was determined using the CAIcal server (http://genomes.urv.es/CAIcal/) (Puigbo et al., 2008). Finally, open reading frame (ORF) prediction for this virophage-like genome was performed using GeneMarkS software (Besemer and Borodovsky, 2005), and the annotation was performed by comparative genomic analyses using BLASTx and BLASTn tools (Altschul et al., 1990).

### Phylogenetic analysis

Alignment of conserved genes was performed using Muscle software (Edgar, 2004). Phylogenetic trees were reconstructed using MEGA6 software using the maximum-likelihood method and the most appropriate substitution model (Tamura et al., 2011).

## Results

### Global diversity estimation of *Megavirales* member- and virophage-related sequences detected in the bioreactor metagenome

**Figure 1:**
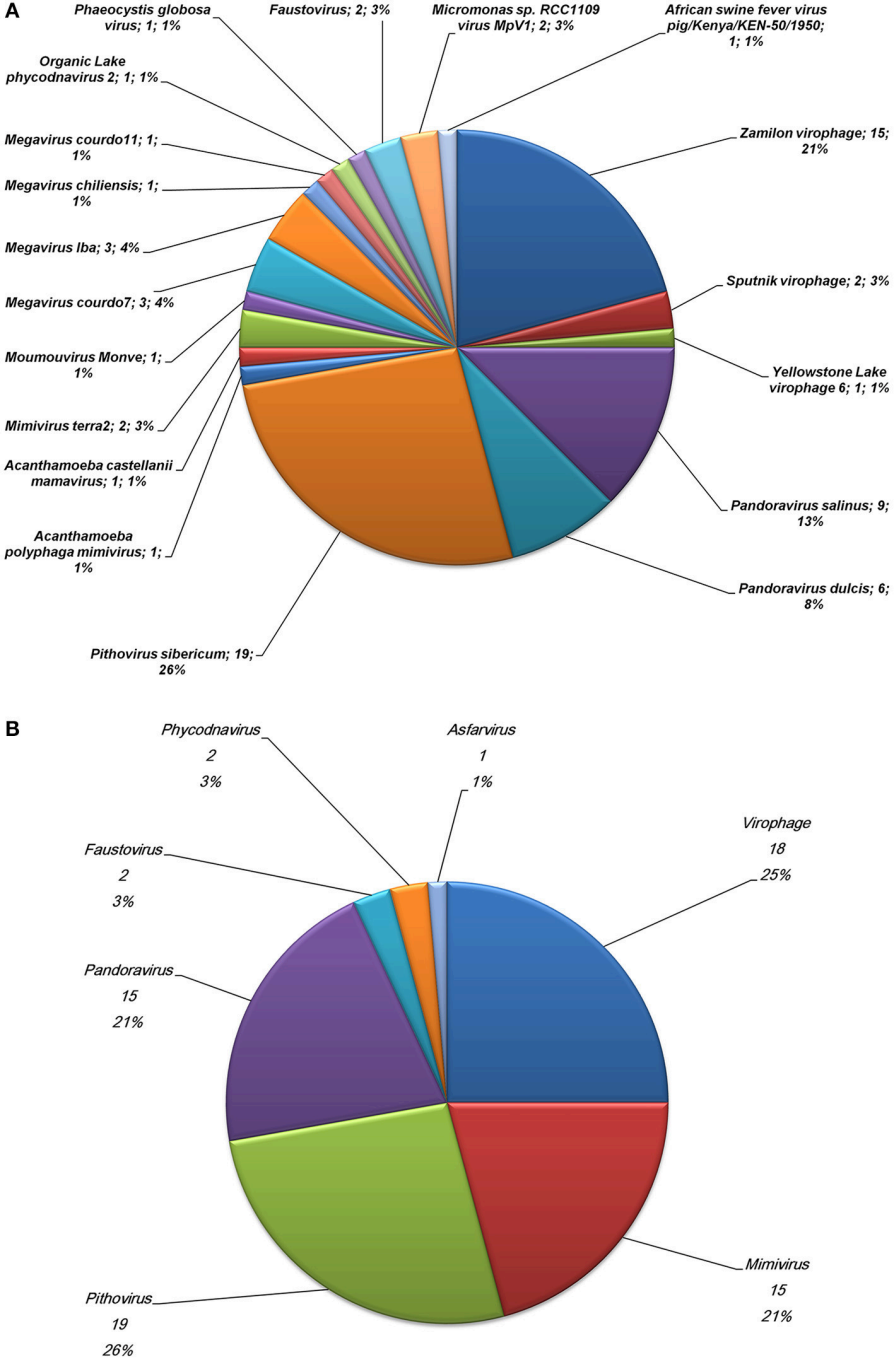
**Relative distribution of *Megavirales* and virophage representatives (A) or viral families (B) found as best hit for contig sequences from the bioreactor metagenome**.

**Figure 2:**
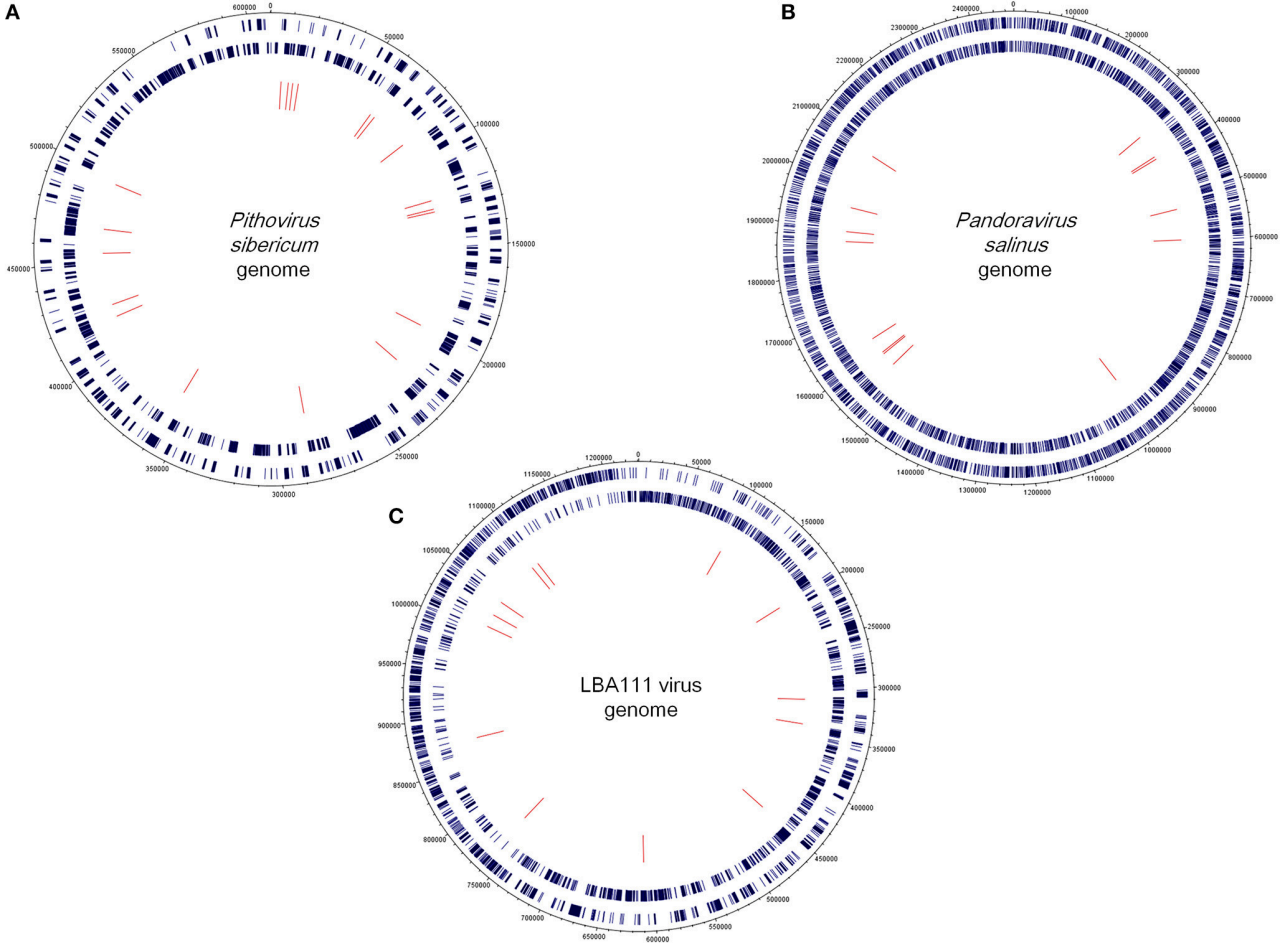
**Circular representations showing the mapping of the bioreactor metagenome contigs on the genomes of *Pithovirus sibericum* (A), *Pandoravirus salinus* (B) and LBA111 virus, a mimivirus of amoeba of lineage C (C)**. Outer rings indicate giant viral open reading frames (ORFs) in sens (outer) and antisense (inner) orientations. Inner ring indicates bioreactor metagenome contigs. Representations were created using DNAPlotter (http://www.sanger.ac.uk/resources/software/dnaplotter/, last accessed June 28, 2015).

The community comprised of megavirus- and virophage-related entities present in a poplar biomass non-aerated bioreactor over the course of a year (Van der Lelie et al., 2012) was assessed through investigating metagenome sequences. Of the 748,672 contig sequences generated by WGS from this bioreactor sample (Van der Lelie et al., 2012), 32,156 (4.2%) showed significant similarities to amino acid sequences from *Megavirales* representatives and virophages. Subsequently, 72 contig sequences (0.01% from the metagenome WGS dataset) were found to have a sequence from megaviruses or virophages as best hit (Figures 1A,B; Supplementary Table S1). These sequences ranged in size from 206 to 773 bp [mean ± standard deviation (SD), 398 ± 113 bp]. Amongst them, 18 (25%) had as best matches virophages and 15 (21%) had as best matches mimiviruses, including 4, 1, and 8 from lineages A, B, and C of amoebal mimiviruses, respectively. Other best matches included sequences from other giant viruses of amoeba such as *P. sibericum* (19 hits); pandoraviruses (15 hits); and recently described faustoviruses (2 hits) (Figures 1A,B; Supplementary Table S1; Figures 2A–C). These best matches were mainly scattered along the giant virus genomes. They included DNA dependent RNA polymerase subunits 1 (from *P. sibericum*) and 2 (from mimiviruses); a DNA polymerase delta catalytic subunit and a VV D5-like helicase (from *P. sibericum*); a GMC oxidoreductase (from *Pandoravirus salinus*); a putative metal-dependent phosphohydrolase, a transcription termination factor and a putative WbbJ acetyltransferase (from mimiviruses); a cysteine desulfurase and a putative DNA repair protein (from faustoviruses); and 4 ankyrin repeat-containing proteins (from mimiviruses and *P. salinus*). In addition, 28 hypothetical proteins from amoebal giant viruses were found as best hits. Overall, 47 bioreactor metagenome contigs were only found to have homologs in the genomes from *P. sibericum* (in 15 cases), pandoraviruses (in 9 cases), mimiviruses (in 6 cases), or virophages (in 17 cases). Otherwise, two best hits were sequences from phycodnaviruses and one best hit was a sequence from an asfarvirus (a 220 kDa polyprotein). Of note, another metagenome contig sequence found as best hit a fatty acid betaoxidation-related protein from *A. castellanii*.

Regarding sequences from virophages found as best hit, 15 (83%) were from Zamilon, 2 were from Sputnik and 1 was from Yellowstone Lake virophage 6 (Figures 1A,B; Supplementary Table S1; Table 2). Moreover, contigs from the bioreactor metagenome that showed the greatest homology to our viral sequence set were highly homologous to Zamilon predicted proteins, amino acid identity being 92% on average and ranging from 82 to 99%.

**Table 2:** **Abundance of virophage sequences identified (BLAST with a threshold of 10^−3^ for the *e*-value) in the Bioreactor metagenome**.

**Virophage strain**	**GenBank Accession no**.	**Year**	**Location**	**Associated virus**	**Protistan host**	**Size (bp)**	**Genome**			**References**
							**Number of ORFs**	**G+C (%)**	**No. of sequences with a virophage gene as best hit**	
Sputnik	EU606015	2008	A cooling tower in Paris, France	Mamavirus	*Acanthamoeba polyphaga*	18,343	21	27	225	La Scola et al., 2008
Mavirus	NC_015230	2010	Coastal waters of Texas	*Cafeteria roenbergensis* virus	Marine phagotrophic flagellate	19,063	20	30.3	17	Fischer and Suttle, 2011
Organic Lake virophage (OLV)	HQ704801	2011	Organic Lake, a hypersaline meromictic lake in Antarctica	Organic Lake Phycodnavirus	Prasinophytes (phototrophic algae)	26,421	24	36.5	616	Yau et al., 2011
*Phaeocystis globosa* virus virophage	NC_021333	2013	Dutch coastal waters (southern North Sea)	*Phaeocystis globosa* virus PgV-16T	Algae	19,527	16	35.8	7	Santini et al., 2013
Zamilon	NC_022990	2014	Tunisia	Mont1 virus (mimivirus)	*Acanthamoeba polyphaga*	17,276	20	29.7	46	Gaia et al., 2014

*bp, base pairs; ORF, open reading frame*.

### Partial genome of a zamilon-like virophage

**Figure 3:**
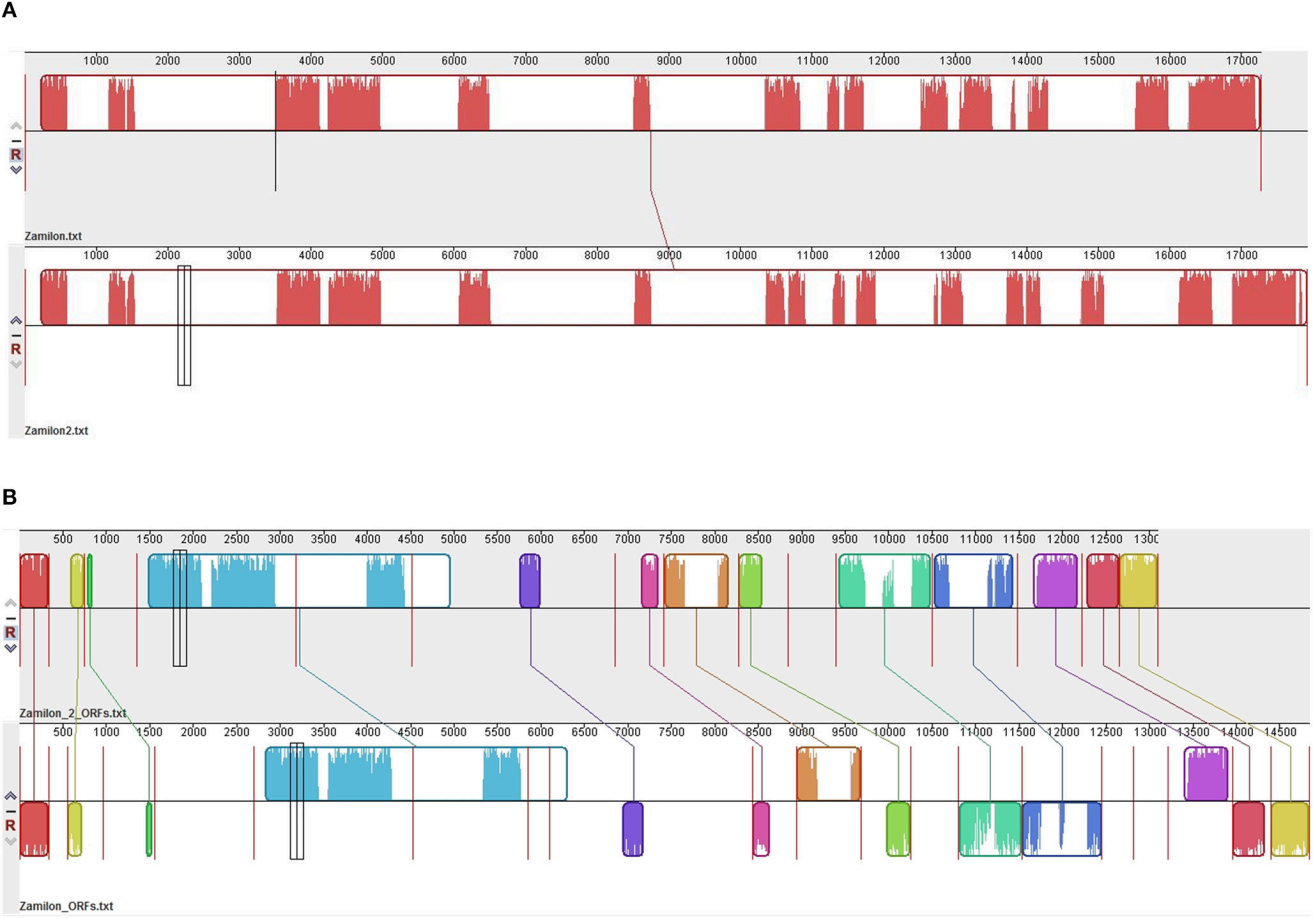
**Comparison of the ORFs (A) and genomes (B) from Zamilon and Zamilon 2**. Comparisons were performed using Mauve software (Darling et al., 2004).

The 15 metagenome sequences that best matched a Zamilon virophage sequence ranged in size from 229 to 534 bp (mean ± SD, 414 ± 107 bp). Their assembly generated a partial genome from a putative new virophage we named Zamilon 2. This partial genome was 6716 bp long, and contained 15 partial predicted ORFs from Zamilon (Table 3; Figures 3A,B). These ORF fragments ranged in size from 57 to 1341 bp (mean ± SD, 432 ± 329). Overall, the partial genome corresponded to 39% of the Zamilon genome; its G+C content was 32%.

**Table 3:** **Zamilon 2 open reading frames and their closest homologs predicted in Zamilon and Sputnik genomes**.

**Zamilon 2 ORF**	**Zamilon**				**Sputnik**				
	**ORF**	**Query cover (%)**	***E*-value**	**Amino acid identity (%)**	**ORF**	**Query cover (%)**	***E*-value**	**Amino acid identity (%)**	**Gene product**
gp01	gp01	81	5,00E-33	91	V15	15	0.001	65	Hypothetical protein
gp03	gp03	27	4,00E-15	82	–	–	–	–	Hypothetical protein
gp04	gp04	6	4,00E-04	92	–	–	–	–	Putative transposase
gp06	gp06	77	0.0	83	V20	77	0.0	77	Capsid protein
gp07	gp07	31	4,00E-68	87	V21	32	9,00E-50	66	Hypothetical protein
gp09	gp09	9	2,00E-42	99	V13	9	1,00E-23	63	DNA replication protein
gp10	gp10	40	2,00E-37	90	V11	36	2,00E-11	45	Hypothetical protein
gp11	gp11	47	2,00E-22	93	V10	7	3,00E-04	64	Putative integrase
gp12	gp12	47	2,00E-44	98	V9	47	8,00E-37	74	Hypothetical protein
gp13	gp13	4	0.82	78	–	–	–	–	Hypothetical protein
gp14	gp14	42	8,00E-58	88	V7	62	3,00E-57	88	Hypothetical protein
gp15	gp15	31	2,00E-26	87	V6	37	2,00E-27	81	Collagen-like protein
gp18	gp18	62	3,00E-78	98	V3	61	2,00E-64	82	DNA packaging ATPase
gp19	gp19	62	3,00E-51	97	V12	61	5,00E-15	42	Hypothetical protein
gp20	gp20	99	2,00E-37	96	V1	16	2,00E-11	80	Hypothetical protein

*ORF, open reading frame*.

### Comparative analyses between zamilon-related and zamilon genomes

Fifteen ORFs were predicted for the Zamilon 2 partial genome. All but two matched confidently with fragments from Zamilon virophage genes based on BLASTn and BLASTx analysis, and showed with them a homology = 78% (Table 3). Mean ± SD nucleotide and amino acid identities for these 15 Zamilon 2 ORFs with their Zamilon counterparts were 89 ± 4% (range, 81–96%) and 91 ± 6% (range, 78–99%), respectively. These ORFs included a putative transposase (gp04), a capsid protein (gp06), a DNA replication protein (gp09), a putative integrase (gp11), a collagen-like protein (gp15), a packaging ATPase (gp18), and hypothetical proteins (gp01, gp03, gp07, gp10, gp12, gp13, gp14, gp19, and gp20) (Table 3). Amongst these sequences, fragments from three conserved core genes were identified. They included fragments encoding a major capsid protein (MCP) (gp06), a DNA replication protein (HEL) (gp09), and a DNA packaging protein (ATPase) (gp18). Both gp06 and gp18 sequences were those with the higher significant similarity to (83 and 62%, respectively) and coverage of (77 and 98%, respectively) Zamilon virophage proteins, whereas the gp09 sequence only covered a very small fragment of its Zamilon counterpart gene (9%). A complete sequence for Zamilon 2 genes, based on their homology to Zamilon genes, was only obtained in two cases, for the gp01 and gp20 genes; the former gene contains two stop codons and the latter contains one. The presence of these stop codons would have deserved being checked to ensure that it did not result from sequencing errors, but new sequencing was not possible here as we only dealt with the metagenome sequences and the sample from which they were generated was not available to us. Overall, according to these findings, Zamilon 2 appeared as closely related to the Zamilon virophage.

### Phylogenetic analysis

**Figure 4:**
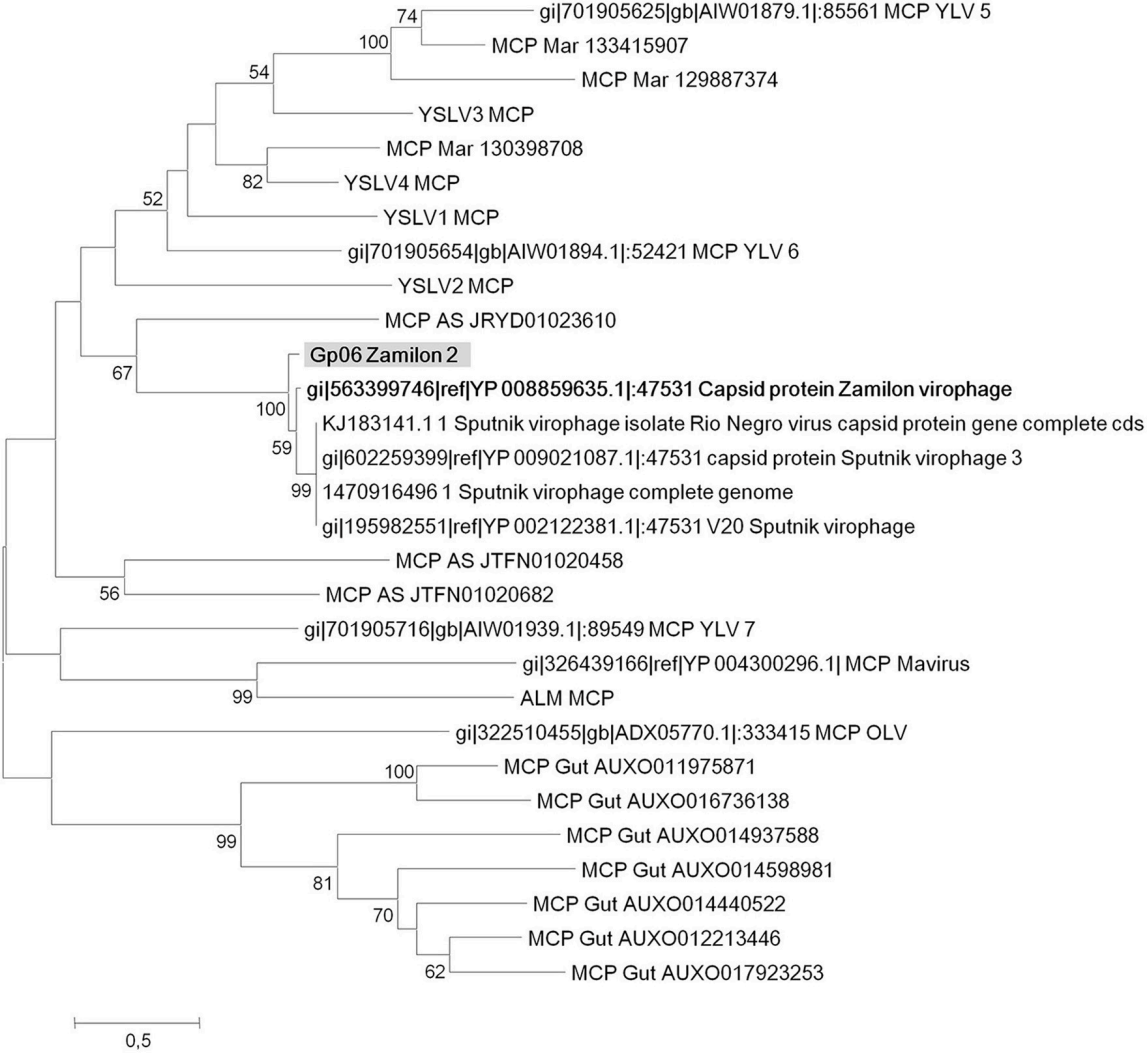
**Phylogenetic reconstruction based on Zamilon 2 gp06 gene product and its homologs (major capsid protein)**. Maximum likelihood-based phylogenetic analysis was performed using MEGA 6.0 software. Bootstrap values >50% are labeled on the tree. ALM, Ace Lake Mavirus; MCP, major capsid protein; YSLV, Yellowstone Lake Virophage; OLV, Organic Lake virophage.

**Figure 5:**
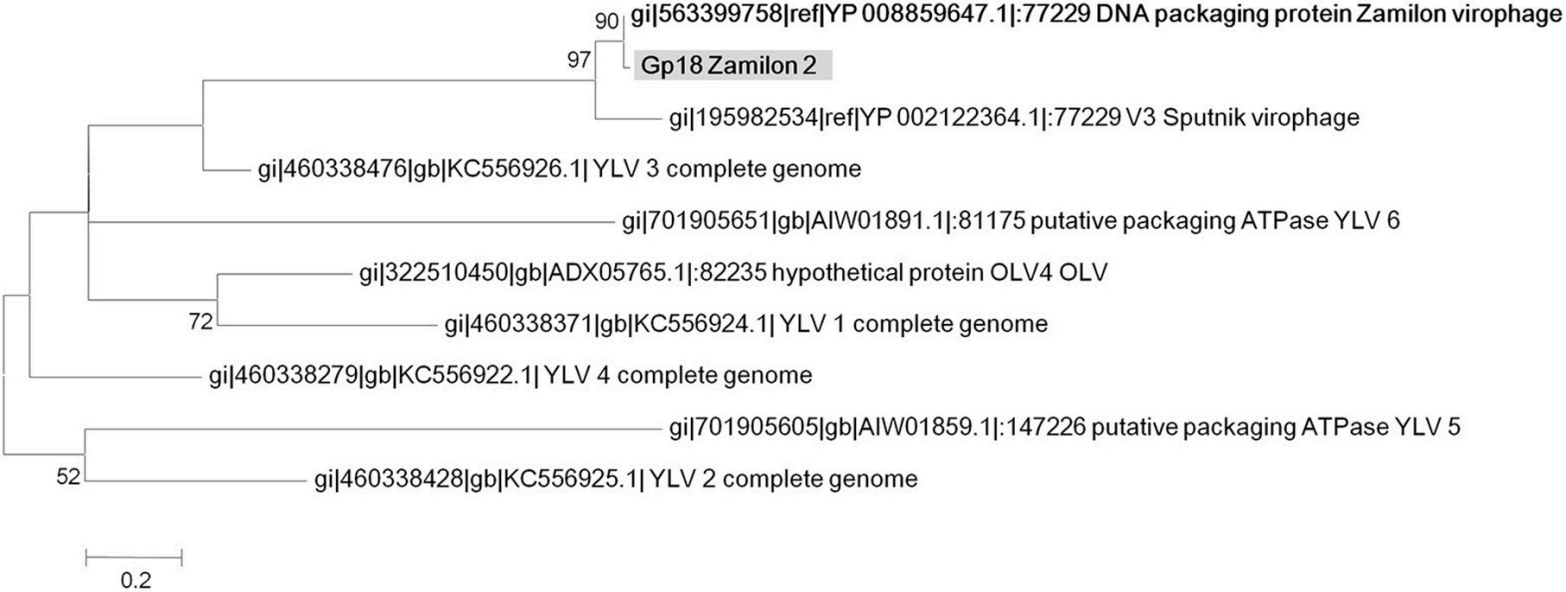
**Phylogenetic reconstruction based on Zamilon 2 gp18 gene product and its homologs (DNA packaging ATPase)**. Maximum likelihood-based phylogenetic analysis was performed using MEGA 6.0 software. Bootstrap values >50% are labeled on the tree. ALM, Ace Lake Mavirus; YSLV, Yellowstone Lake Virophage; OLV, Organic Lake virophage.

**Figure 6:**
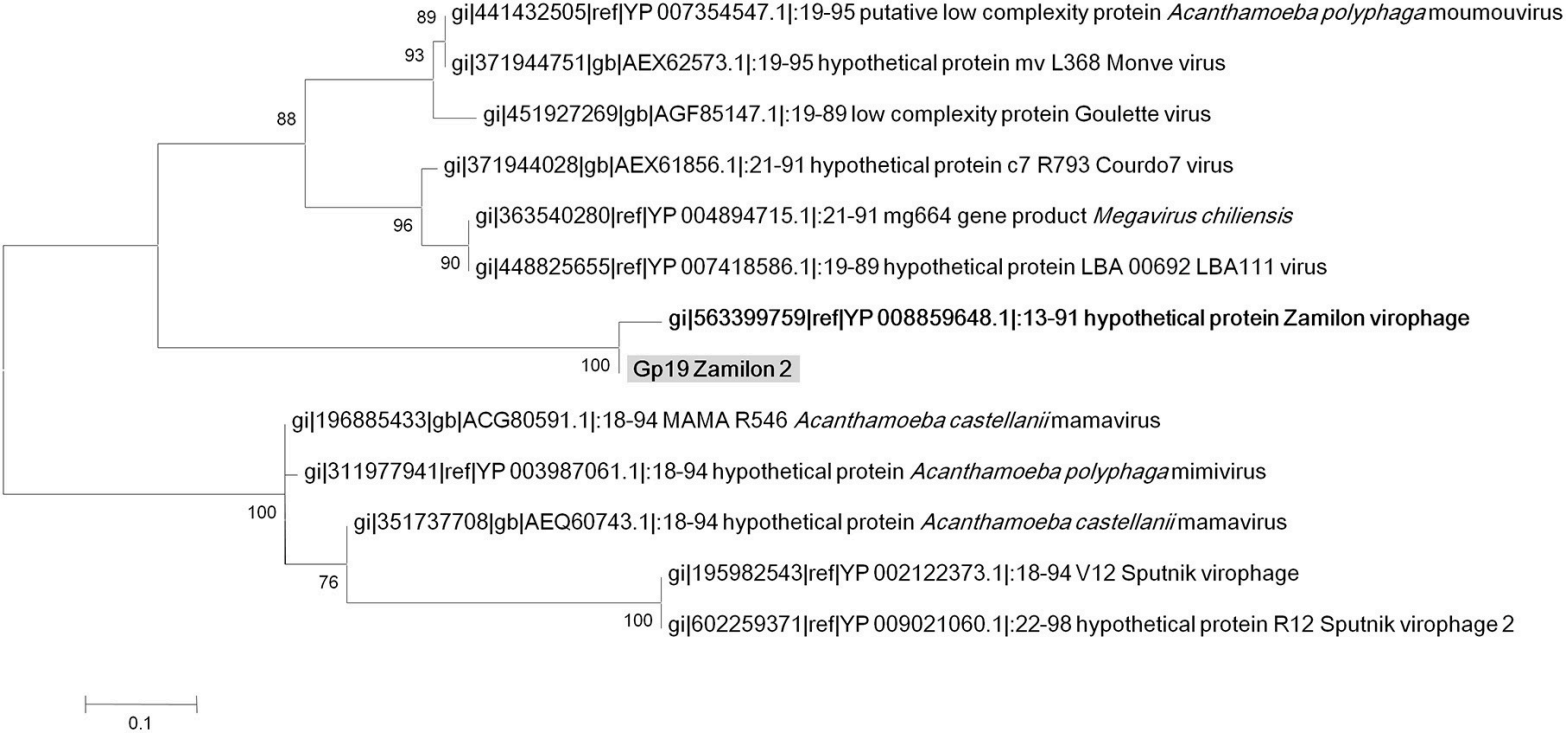
**Phylogenetic reconstruction based on Zamilon 2 gp19 gene product and its homologs**. Maximum likelihood-based phylogenetic analysis was performed using MEGA 6.0 software. Bootstrap values >50% are labeled on the tree. ALM, Ace Lake Mavirus; YSLV, Yellowstone Lake Virophage; OLV, Organic Lake virophage.

**Figure 7:**
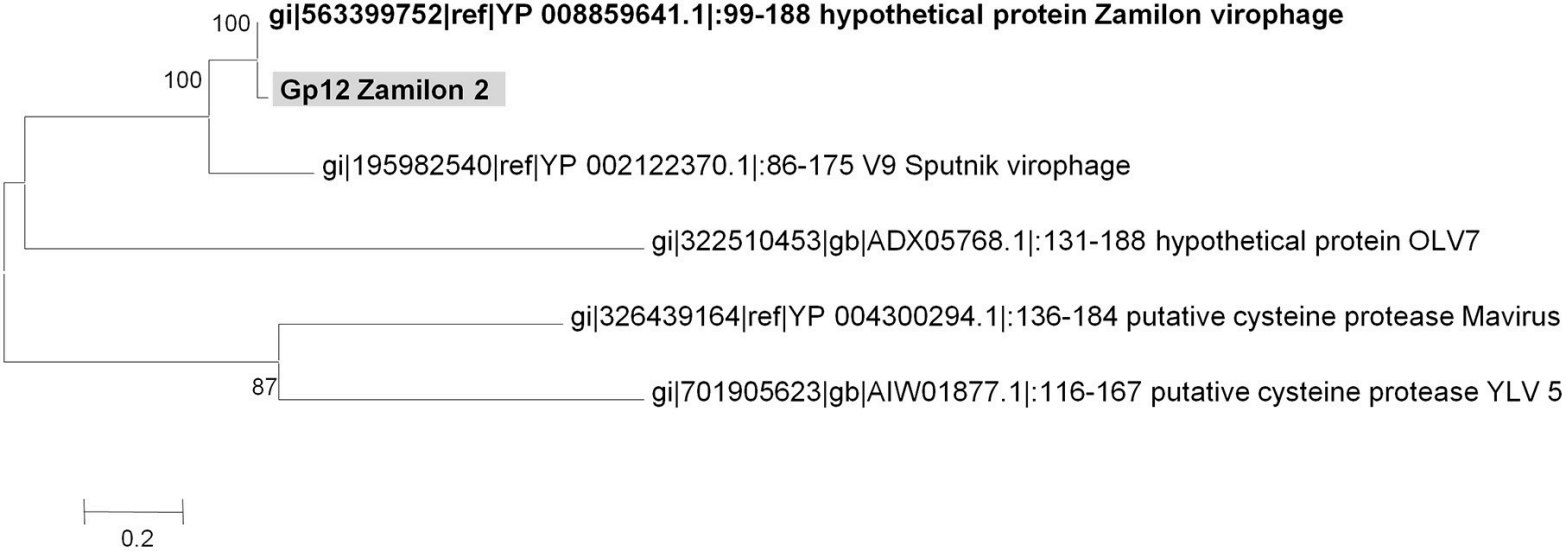
**Phylogenetic reconstruction based on Zamilon 2 gp12 gene product and its homologs**. Maximum likelihood-based phylogenetic analysis was performed using MEGA 6.0 software. Bootstrap values >50% are labeled on the tree. ALM, Ace Lake Mavirus; YSLV, Yellowstone Lake Virophage; OLV, Organic Lake virophage.

Four virophage genes, including core genes encoding MCP (gp06) and packaging ATPase (gp18), and for which sequence coverage with other virophage sequences was sufficient for the purpose of phylogeny reconstruction were analyzed. Phylogeny based on MCPs was congruent with that recently described by Yutin et al. (2015) and showed that capsid generated from the bioreactor metagenome was most closely related to the Zamilon virophage (Figure 4). A phylogenetic tree based on putative packaging ATPase (gp18 in the Zamilon 2 partial genome) (Figure 5) showed that Zamilon 2 is clustered with Zamilon, within the Sputnik/Zamilon virophage group, and that these two viruses comprise a sister group to the Sputnik virophage. Regarding phylogeny reconstruction based on the Zamilon gp19 gene homolog, its topology was consistent with that observed previously, Zamilon being clustered with amoebal mimiviruses of lineages B and C, whereas Sputnik virophages were clustered with amoebal mimiviruses of lineage A (Figure 6; Gaia et al., 2014). Finally, the topology of the phylogenetic tree based on the Zamilon gp12 hypothetical protein showed a cluster comprised by Zamilon 2 and Zamilon as a sister group of the Sputnik virophage (Figure 7).

Taken together, the results from comparative genomics and phylogenetic analyses indicated that the partial genome assembled here was that of a putative new Zamilon-like virophage.

## Discussion

We assembled here one third of a Zamilon-like genome (that we named Zamilon 2) by using metagenomic sequences generated from a poplar wood chips bioreactor (Van der Lelie et al., 2012). Consistently, we found sequences related to various *Megavirales* members, most of which being giant viruses of *Acanthamoeba* including mimiviruses, the known Zamilon hosts. Comparative genomics and phylogenetic analyses based upon 4 genes clearly showed that the Zamilon 2 genome was that from a Zamilon-close relative, and Zamilon 2 and Zamilon comprised a sister group to Sputnik virophages. Sequences encoding a Zamilon-like MCP protein had been detected in this metagenome, but additional virophage genes were not described as only sequences matching capsid proteins were searched for and other contigs than the one harboring the capsid gene were not analyzed (Yutin et al., 2015). Virophages- and *Megavirales*-related sequences detected in this bioreactor metagenome may have been generated from the poplar woody material originally collected, although it cannot be excluded they were generated from solution used to humidify this material. Noteworthy, to our best knowledge, we detected here for the first time in metagenomes sequences related to *P. sibericum*, whereas pandoravirus-related sequences were recently detected from various soil samples collected worldwide (Kerepesi and Grolmusz, 2015) and faustovirus-related sequences were detected in Mississippi ponds and sera from healthy Egyptians (Reteno et al., 2015).

Owing to a tremendous advance of sequencing technologies, metagenome sequencing has proved extremely powerful over recent years in studying viral communities and virus prevalence in numerous environments, animals and in healthy and diseased humans (Mokili et al., 2012). Moreover, some partial and even full-length viral genomes could be assembled from metagenomic sequences (Smits et al., 2014). Regarding virophages, metagenomic datasets allowed these viruses to be identified and located in various environments, and full-length and partial genomes could be reconstructed (Yau et al., 2011; Zhou et al., 2013, 2015; Zablocki et al., 2014; Yutin et al., 2015). Previous findings and this work suggest that virophages are widespread in our biosphere. Zhou et al. described, through the analysis of metagenomic datasets, that sequences related to these viruses were detected in metagenomes generated from various environmental samples consisting of freshwater or marine water samples or soil samples collected worldwide in geographical areas with various climates (Zhou et al., 2013). Yutin et al. also found virophage-related sequences in metagenomes generated from activated sludge, freshwater sediment, marine water, and wastewater (Yutin et al., 2015). Hence, virophage distribution appears consistent with that of mimiviruses and *Acanthamoeba* spp, which are both known to be widespread in our biosphere (Thomas and Greub, 2010; Colson and Raoult, 2012; Pagnier et al., 2013). Moreover, phylogeny reconstruction based on MCP sequences from virophages clearly indicates that some virophages from various sources are related (Yutin et al., 2015; Figure 4). Regarding Zamilon virophages, Zamilon was isolated from water in Northern Africa, whereas Zamilon 2 sequences were obtained from a bioreactor in Northern America (Gaia et al., 2014). Similar observations were made for mimiviruses, for which close relatives were isolated from different samples collected on different continents (Yoosuf et al., 2012, 2014).

Phenotypically, Zamilon shares the same hosts with Sputnik virophages, but is distinguished by its host range that allows its replication with only two of the three lineages of amoebal mimiviruses (Gaia et al., 2014). Thus, mimiviruses of lineage A, for instance the original Mimivirus strain, do not support Zamilon replication. This feature deserves additional studies, which are currently on-going in our laboratory, and the availability of other full-length or partial genomes, such as the one reconstructed here, may provide some clues for its understanding. It is worth noting that the mimivirus-like sequences detected here were related to mimiviruses classified in the three lineages A, B, and C.

Evidence has accumulated for mimiviruses, including their recent isolation from patients presenting pneumonia, that suggest their involvement in such a syndrome (Saadi et al., 2013a,b). Hence, this further raises the questions of the association of mimivirus virophages with humans. The worldwide distribution of virophages and their likely non-negligible prevalence in our biosphere suggest that mammals, including humans, are potentially commonly exposed to virophages. To date, Sputnik 2 virophage has been isolated from a contact lens rinse liquid used by a keratitis patient, in association with Lentillevirus and *Acanthamoeba polyphaga* (Cohen et al., 2011). In addition, antibodies to the Sputnik virophage were detected in two Laotian patients with unexplained fever, and seroconversion was observed in one of the two cases (Parola et al., 2012). It is also worth noting that virophage-related sequences have been detected in metagenomes generated from rumen (Yutin et al., 2015) and human gut (Zhou et al., 2013).

In summary, our findings expand current knowledge on the diversity of virophages. Further studies are needed to gain a better knowledge of their tropism and prevalence in our biosphere and in humans.

### Conflict of interest statement

The authors declare that the research was conducted in the absence of any commercial or financial relationships that could be construed as a potential conflict of interest.
