# Supplementary material for: A New Zamilon-like Virophage Partial Genome Assembled from a Bioreactor Metagenome
Source: Front Microbiol. 2015 Nov 27;6:1308. doi: 10.3389/fmicb.2015.01308 (PMC4661282; doi:10.3389/fmicb.2015.01308)
Supplement: Supplementary file 2 [file DataSheet1.DOCX]

**Supplementary material**

**Supplementary Table S1.** Best tBLASTn hits from *Megavirales* representatives and virophages for bioreactor metagenome contigs.
